# circ_NRIP1 is oncogenic in malignant development of esophageal squamous cell carcinoma (ESCC) via miR-595/SEMA4D axis and PI3K/AKT pathway

**DOI:** 10.1186/s12935-021-01907-x

**Published:** 2021-05-06

**Authors:** Shifan Zhou, Zhizhong Guo, Chaofeng Zhou, Yu Zhang, Sai Wang

**Affiliations:** 1 Henan University of Chinese Medicine, No.156 Jinshui East Road, Zhengzhou, 450046 Henan China; 2 Department of Oncology, The Second Affiliated Hospital of Henan University of Chinese Medicine, No.6 Dongfeng Road, Jinshui District, Zhengzhou, 450002 Henan China

**Keywords:** Circ_NRIP1, MiR-595, SEMA4D, ESCC

## Abstract

**Background:**

The hsa_circ_0004771 derived from NRIP1 (called circ_NRIP1) is a recently identified oncogenic circRNA. Here, we intended to investigate the role and mechanism of circ_NRIP1 in esophageal squamous cell carcinoma (ESCC), a prevalent and aggressive type of esophageal cancer.

**Methods:**

Expression of circ_NRIP1, miRNA-595-5p (miR-595) and semaphorin 4D (SEMA4D) was detected by RT-qPCR and western blotting. Cell growth was assessed by colony formation assay, MTS assay, flow cytometry, and xenograft experiment; migration and invasion were evaluated by transwell assay and western blotting. Dual-luciferase reporter assay identified the relationship among circ_NRIP1, miR-595 and SEMA4D. Western blotting measured phosphatidylinositol-3-hydroxykinase (PI3K)/AKT pathway-related proteins.

**Results:**

Expression of circ_NRIP1 was upregulated in ESCC tissues and cells. Knockdown of circ_NRIP1 could enhance apoptosis rate and E-cadherin expression, but suppress colony formation, cell viability, migration, invasion, and snail expression in KYSE30 and KYSE450 cells, as well as retarded tumor growth in mice. The suppressive role of circ_NRIP1 knockdown in cell growth, migration and invasion in vitro was abated by blocking miR-595; meanwhile, miR-595 overexpression elicited similar anti-tumor role in KYSE30 and KYSE450 cells, which was abrogated by restoring SEMA4D. Notably, circ_NRIP1 was a sponge for miR-595, and SEMA4D was a target of miR-595. Besides, PI3K/AKT signal was inhibited by circ_NRIP1 knockdown and/or miR-595 overexpression via indirectly or directly regulating SEMA4D.

**Conclusion:**

circ_NRIP1 functioned as an oncogene in ESCC, and modulated ESCC cell growth, migration and invasion both in vitro and in vivo via targeting miR-595/SEMA4D axis and inhibiting PI3K/AKT signaling pathway.

**Supplementary Information:**

The online version contains supplementary material available at 10.1186/s12935-021-01907-x.

## Background

Esophageal squamous cell carcinoma (ESCC) is the most prevalent and aggressive type of esophageal cancer ranking the sixth lethal types of cancer worldwide, especially in Eastern Asia [1, 2]. Multimodal treatment options for ESCC include surgery, chemotherapy, and radiotherapy; however, the prognosis of ESCC was rather disappointing than its counterpart with esophageal adenocarcinoma [3]. Furthermore, its presentation and diagnosis usually occur late, and early diagnosis is associated with much higher 5 year survival [4]. In consideration that ESCC is regarded as epigenetic abnormal disease, including noncoding RNAs (ncRNAs) regulation [5], it is of utmost urgency to identify novel informative ncRNAs in the initiation, progression, and metastasis of ESCC.

Circular RNAs (circRNAs) are an emerging class of ncRNAs, which are covalently closed. It is reported that the biogenesis and biological functions of circRNAs have been discussed in cancers including ESCC [6], as well as their molecular diagnostic values. The circRNA profiles have also been surveyed in plasma [7] and tissues [8] from ESCC patients, as well as ESCC cell lines [9]. Mechanically, competitive endogenous RNAs (ceRNAs) network is a classic mechanism of dysregulated circRNAs in ESCC [10]. The hsa_circ_0004771 is a recently identified oncogenic circRNA [11–13], which is derived from exon 2 and exon 3 of nuclear receptor interacting protein 1 (NRIP1), thus also named as circ_NRIP1. However, the research of circ_NRIP1 remains in initial stage, especially in ESCC.

MicroRNAs (miRNAs) are a well-defined type of ncRNAs with appropriate 22 nucleotides. The differential roles of miRNAs have been reviewed in the pathogenesis and treatment of esophageal cancer including ESCC [14, 15], as well as their prognostic and therapeutic role [16]. Activation of phosphatidylinositol-3-hydroxykinase (PI3K) and AKT are reported to occur in many cancers, such as breast, ovarian, pancreatic, and esophageal cancer [17]. Moreover, miRNAs are closely interacted with PI3K/AKT signaling pathway in esophageal cancer [18]. MiRNA-595-5p (miR-595) has been demonstrated to be involved in tumorigenesis of several cancers through positively or negatively regulating malignant progression [19, 20]. However, the relationship among miR-595, PI3K/AKT signal and ESCC remains unrevealed.

Semaphorin 4D (SEMA4D) is one member of semaphorin family [21], which is known as membrane semaphorins. In this study, we aimed to detect the expression of circ_NRIP1 in ESCC patients, and its role in ESCC cell growth, migration and invasion both in vitro and in vivo; furthermore, a novel ceRNA pathway associated with circ_NRIP1, miR-595 and SEMA4D was to figure out in the malignant cell progression, as well as PI3K/AKT signaling pathway.

## Materials and methods

### Clinical tissue collection

The tumor tissues and adjacent (≥ 5 cm away from primary lesion) normal tissues were acquired from 42 ESCC patients during the primary esophagectomy at The Second Affiliated Hospital of Henan University of Chinese Medicine. Patients who had received preoperative chemoradiotherapy were excluded, and all patients signed the informed consent before surgery. The clinical information of ESCC patients are also shown in Table 1. This study was approved by the Medical Ethics Committee of The Second Affiliated Hospital of Henan University of Chinese Medicine. The tissues were snap-frozen in liquid nitrogen prior to total RNA isolation and protein extraction.

**Table 1:** The clinical information of ESCC patients

Characteristics	Groups	Numbers (n = 42)	circ_NRIP1 expression		
			High	Low	*P*-value
Age	≤ 63	23	11 (47.8%)	12 (52.2%)	0.652
	> 63	19	10 (52.6%)	9 (47.4%)	
Gender	Male	24	13 (54.2%)	11 (45.8%)	0.723
	Female	18	8 (44.4%)	10 (55.6%)	
TNM Stage	pT1-pT2	19	2 (10.5%)	17 (89.5%)	0.016
	pT3-pT4	23	19 (82.6%)	4 (17.4%)	
Cancer location	Upper	7	4 (57.1%)	3 (42.9%)	0.541
	Middle	25	13 (52.0%)	12 (48.0%)	
	Lower	10	4 (40.0%)	6 (60.0%)	
Treatment	No	42	21 (50.0%)	21 (50.%)	

### Cell culture

Human ESCC cell lines including, KYSE30 (#0188) and KYSE450 (#1430) were from Japanese Collection of Research Bioresources (JCRB; NIBIO, Ibaraki, Osaka, Japan), and the normal esophagus epithelial cell line HET-1A (#2692) was from American Type Culture Collection (ATCC; Manassas, VA, USA). The KYSE30 cells were cultured in DMEM (Hyclone, Logan, UT, USA) with 2% fetal bovine serum (FBS; Hyclone), and KYSE30 cells were cultured in Ham’s F12 and RPMI1640 medium (1 to 1 mix; Hyclone) with 2% FBS, HET-1A cells were in DMEM plus 10% FBS. All the cells were incubated in a humidified atmosphere of 5% CO_2_ at 37 °C.

### Reverse transcription-quantitative polymerase chain reaction (RT-qPCR)

Total RNA was extracted with TRIzol reagent (Invitrogen, Carlsbad, CA, USA), and miRNA was extracted with a mirVanaTM miRNA kit (Ambion, Austin, TX, USA). The cDNA was synthesized using Superscript First-Strand Synthesis System (Invitrogen), followed by qPCR using SYBR Premix Ex Taq II kit (Takara, Dalian, China). The 2^−ΔΔCt^ method determined the relative levels of target RNA expression; glyceraldehyde-phosphate dehydrogenase (GAPDH) was the endogenous control for circ_NRIP1, SEMA4D and NRIP1, and U6 snoRNA (U6) was for miR-595. The special sequence of primers was circ_NRIP1 (5′-CTCCGGATGACATCAGAGCT-3′ and 5′-TCACAATCCAAACACTTCCGT-3′), NRIP1 (5′-GGAAGTGTTTGGATTGTGAGCT-3′ and 5′-CCCTGTCCTCCTTCAGTCAA-3′), miR-595 (5′-AGTGTGCCGTGGTGTG-3′ and 5′-GAACATGTCTGCGTATCTC-3′), SEMA4D (5′-AGCTCTGCACAAAGCCATCAGC-3′ and 5′-CCAGCATAGACAAACCTGTTGCC-3′), GAPDH (5′-AGAAGGCTGGGGCTCATTTG-3′ and 5′-AGGGGCCATCCACAGTCTTC-3′), and U6 (5-AAAGCAAATCATCGGACGACC-3′ and 5′-GTACAACACATTGTTTCCTCGGA-3′). For RNase R treatment, total RNA from KYSE30 and KYSE450 cells was incubated with 3 U/μg RNase R (Epicentre Technologies, Madison, WI, USA) for 20 min at 37 °C. The mock RNA was without RNase R treatment.

### Cell transfection

KYSE30 and KYSE450 cells were administrated with siRNA against circ_NRIP1 (called as si-circ_RIP1;5′ UCACAAUCCAAACACUUCCGU-3′), miR-595 mimic (5′-GAAGUGUGCCGUGGUGUGUCU-3′), anti-RNAs against miR-595 (named as anti-miR-595; 5′-AGACACACCACGGCACACUUC-3′) or SEMA4D-overexpression vector using Lipofectamine 2000 (Invitrogen) according to the manufacturer’s instructions, as well as their negative controls including si-NC (5′-AAUUCUCCGAACGUGUCACGU-3′), miR-NC mimic (5′-UUUGUACUACACAAAAGUACUG-3′), anti-miR-NC (5′-UCUACUCUUUCUAGGAGGUUGGA-3′) and pcDNA3.1 (−) empty vector (simplified as pcDNA). The oligonucleotides were synthesized by GenePharma (Shanghai, China), and the whole sequences of SEMA4D were cloned in pcDNA vector (Invitrogen).

### Cell viability assay and colony formation assay

Cell viability of KYSE30 and KYSE450 cells after transfection was determined by MTS method using CellTiter 96 AQueous One Solution Cell Proliferation Assay (Promega, Madison, WI, USA). Briefly, transfected cells in 96-well plate were added with 10% MTS after transfection for 0, 1, 2 and 3 days. With 4 h-incubation of MTS, the optical density (OD) values were measured on SpectraMax Microporous plate reader (Molecular Devices, Shanghai, China) at 490 nm.

After transfection for 1 day, KYSE30 and KYSE450 cells were passaged in 6-well plate at density of 1 000 cells/well in complete culture medium for another 15 days. The fresh medium was replaced every three days. On the last day, cells were fixed with 95% methanol for 10 min and stained with 0.1% crystal violet for 10 min. The cell colonies were captured and counted under microscope.

### Cell apoptosis analysis

After transfection for 1 day, apoptotic cells of KYSE30 and KYSE450 cells were stained by Annexin V-fluorescein isothiocyanate (FITC) and propidium iodide (PI) double staining method using Annexin V-FITC Apoptosis Detection Kit (TransGen, Beijing, China). The stained cells were analyzed on flow cytometry (FCM), and apoptotic rate was detected in Annexin V-FITC/PI quadrants.

### Transwell migration and invasion assays

Transwell filter (8-µm pore size, Corning, New York, NY, USA) was prepared with or without Matrigel to measure cell invasion and migration, respectively. 5 × 10^5^ transfected cells were plated in 24-well Boyden chamber, and incubated in serum-free medium; the bottom chamber was filled with 400 μL of medium containing 10% FBS. The transwell system was incubated at 37 °C for another 2 days. After moving the cells on the upper membrane, the transferred cells were fixed with 95% ethanol for 10 min, and stained with 0.1% crystal violet for 10 min. The migrated cells and invaded cells were photographed by a microscope at 100 × , and counted using Image J software (National Institutes of Health, USA).

### Western blotting

Total protein in tissues and cells was extracted in 10 × Cell Lysis Buffer (CST, Danvers, MA, USA). After 12 000 × *g* centrifugation for 15 min, the protein content of supernatant was determined by Bradford method. The protein samples were added with cocktail protease inhibitor (Roche, Basel, Switzerland), and subjected to normal western blotting procedures. The immunoreactivity was visualized by ECL assay kit (Millipore, Billerica, MA, USA) and the band intensity was determined by Image J (National Institutes of Health). The primary antibodies against SEMA4D (#82,951), Snail (#3879), GAPDH (#2118), E-cadherin (#3195), p-PI3K (#17366), PI3K (#4292), p-AKT (#2965), AKT (#9272), and the secondary antibody anti-rabbit IgG (HRP) (#7074) were from CST.

### Dual-luciferase reporter assay

The whole length of circ_NRIP1 or 3′ untranslated region of SEMA4D (named as SEMA4D 3′UTR) containing wild type (simplified as wt) or mutant type (simplified as mut) of miR-595 binding sites was inserted in luciferase report vector pGL4 (Promega). KYSE30 and KYSE450 cells were passaged in 24-well plate, and then co-transfected with pGL4 vectors and miR-595 mimic or miR-NC mimic for 2 days. Every group transfection was repeated in three wells. The dual-luciferase reporter assay system (Promega) was used to measure luciferase activities following the guide book.

### Xenograft mice model

Ten male BALB/c mice (5–8 weeks old) provided by Beijing HFK Bioscience Co. Ltd. (Beijing, China), and this animal experiments were approved by the Animal Care and Use Committee of the The Second Affiliated Hospital of Henan University of Chinese Medicine. KYSE450 cells were transfected with circ_NRIP1 silencing vector carrying shRNA against circ_NRIP1 (simplified as sh-circ_NRIP1; 5′-GGAAGTGTTTGGATTGTGA-3′ and 5′-TCACAATCCAAACACTTCC-3′) or control vector carrying negative control sequence (named as sh-NC; 5′-GCTACGATCTGCCCAAGATTT-3′ and 5′-ATCTTAGGCAGATCGUCGCTT-3′), and this technical support was provided by GenePharma; then stably transfected KYSE450 cells were subcutaneously injected into right flanks of nude mice (n = 5) at a density of 1 × 10^7^ cells per mice. The long (a) and short diameters (b) of tumors were first measured using caliper after transplantation for 10 days, and further measured every five days. The tumor volume was calculated using 0.5 × a × b^2^ equation, and tumor weight was recorded using electronic balance on the 30th after transplantation.

### Statistical analysis

Data are shown as the means ± standard error of at least three independent experiments. Graphpad Prism 6.0 software (GraphPad, La Jolla, CA, USA) was utilized to compare the difference between two groups using Student’s *t* test and among multiple groups using analysis of variance. Pearson correlation coefficient assay was utilized to analyze the association among circ_NRIP1, miR-595 and SEMA4D in ESCC tumor tissues.

## Results

### Circ_NRIP1 was a stable highly expressed circRNA in ESCC tissues and cells

**Fig. 1:**
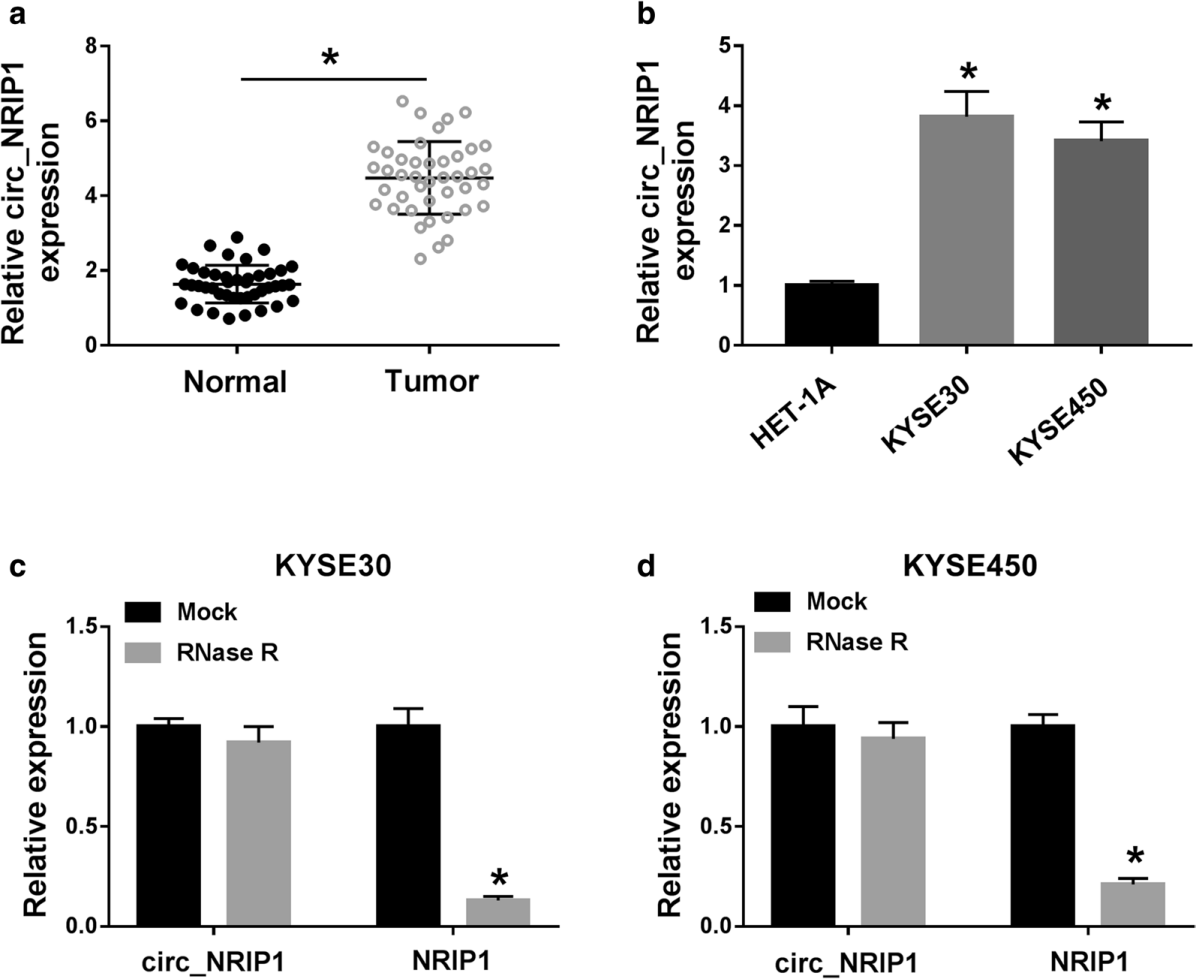
Expression of hsa_circ_0004771 (also called circ_NRIP1) in esophageal squamous cell carcinoma (ESCC) tissues and cells. **a** RT-qPCR detect circ_NRIP1 expression level in 42 paired tumor tissues (Tumor) and adjacent normal tissues (Normal) from ESCC patients. **b** RT-qPCR detect circ_NRIP1 expression level in human ESCC KYSE30 and KYSE450 cells, as well as normal HET-1A cells. **c**, **d** RT-qPCR detect RNA levels of circ_NRIP1 and its host gene NRIP1 in KYSE30 and KYSE450 cells subjected to RNase R, compared to cells mock cells (without RNase R treatment). *P < 0.05

In order to confirm the role of circ_NRIP1 in ESCC, we firstly detect its expression in ESCC patients using RT-qPCR. As shown in Fig. 1a, expression level of circ_NRIP1 was higher in tumor tissues (n = 42) than normal tissues (n = 42) from ESCC patients. Besides, circ_RNIP1 level was associated with TNM stage (Table 1). RT-qPCR also depicted that circ_RNIP1 expression was overall increased in human ESCC cell lines (Additional file 1: Figures S1 and Additional file 2: Figure S2) comparing to that in HET-1A cells, and its expression was the most high in KYSE30 and KYSE450 cells (Additional file 1: Figure S1 and Fig. 1b). The expression of circ_NRIP1 was resistant to RNase R treatment in KYSE30 and KYSE450 cells, which was unlike to its host gene NRIP1 (Fig. 1c, d). These data demonstrated circ_NRIP1 as a stably upregulated circRNA in ESCC tissues and cells.

### Knockdown of circ_NRIP1 suppressed ESCC cell growth, migration and invasion in vitro

**Fig. 2:**
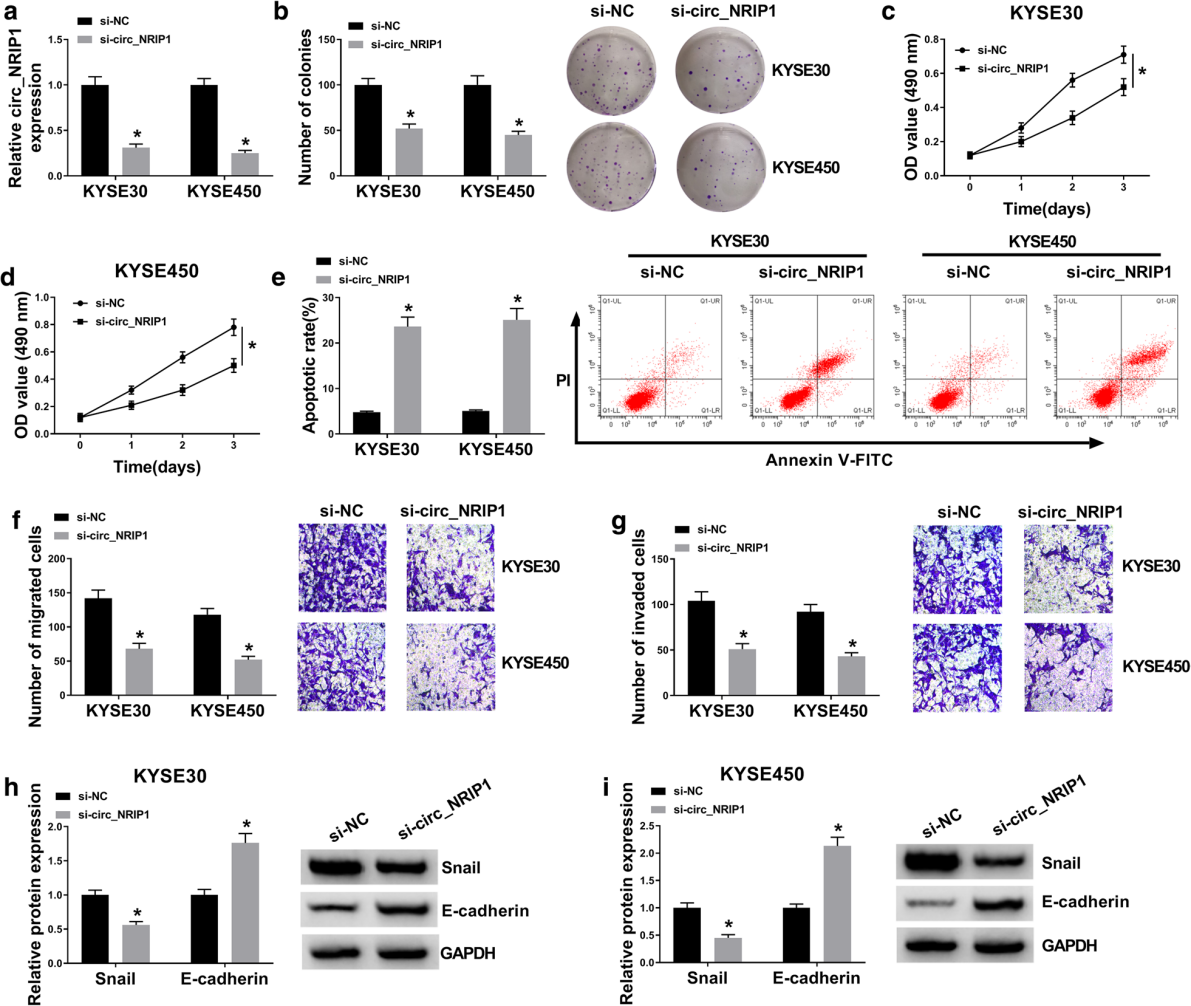
The role of circ_NRIP1 in ESCC cells in vitro. **a** RT-qPCR detect circ_NRIP1 expression level in KYSE30 and KYSE450 cells administrated with siRNA target circ_NRIP1 or negative control sequences (simplified as si-circ_NRIP1 or si-NC). **b** Number of colonies was measured by colony formation assay after transfection. **c**, **d** MTS assay determined optical density (OD) of transfected cells at 490 nm after transfection for 0–3 days. **e** Annexin V-fluorescein isothiocyanate (FITC) and propidium iodide (PI) double staining method and flow cytometry (FCM) analyzed apoptotic rate of transfected cells after transfection for 1 day. **f**, **g** Transwell assays evaluated numbers of migrated cells and invaded cells after transfection for 1 day. **h**, **i** Western blotting examined protein expression of snail and E-cadherin in transfected cells after transfection for 1 day. *P < 0.05

Loss-of-function experiment was conducted to investigate the role of circ_NRIP1 in ESCC cells in vitro. Therefore, KYSE30 and KYSE450 cells were transfected with si-circ_NRIP1 prior to functional analysis versus si-NC transfection group. RT-qPCR was utilized to measure knockdown efficiency, and si-circ_NRIP1 transfection caused a dramatic inhibition of circ_NRIP1 in both KYSE30 and KYSE450 cells (Fig. 2a). Thus, colony formation of KYSE30 and KYSE450 cells was attenuated due to circ_NRIP1 knockdown, as indicated by lowered number of colonies (Fig. 2b); MTS assay indicated a cell viability inhibition in si-circ_NRIP1-transfected KYSE30 and KYSE450 cells (Fig. 2c, d), as accompanied with increased apoptotic rate (Fig. 2e) according to FCM analysis. These results suggested that cell growth of ESCC cells in vitro was suppressed by blocking circ_NRIP1. Next, transwell assay revealed that numbers of migrated cells and invaded cells were reduced after si-circ_NRIP1 transfection (Fig. 2f, g), as well as E-cadherin increase and snail loss (Fig. 2h, i). These results suggested that migration and invasion of ESCC cells in vitro was suppressed by blocking circ_NRIP1. Taken together, circ_NRIP1 knockdown functioned a tumor-suppressive role in ESCC cell malignancy.

### MiR-595 was segregated by circ_NRIP1 in ESCC via target binding

**Fig. 3:**
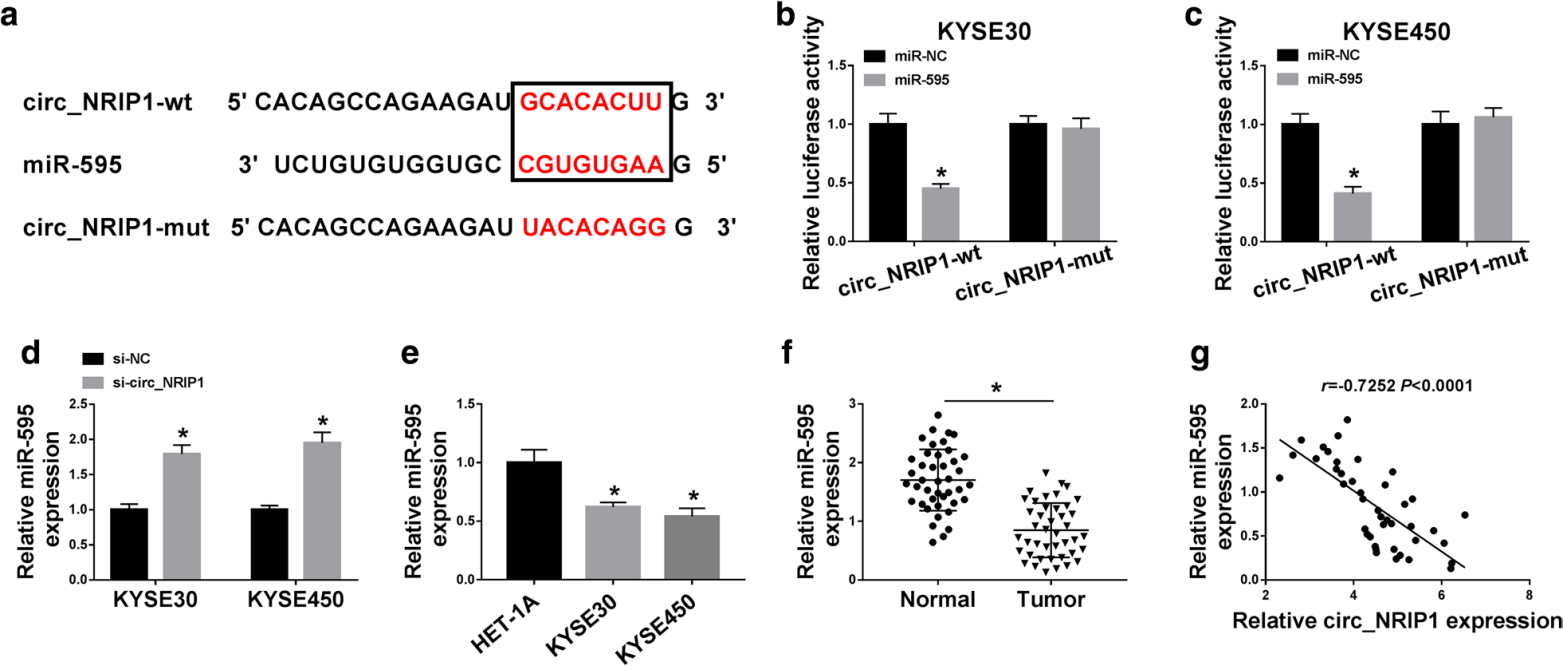
The expression of miRNA-595-5p (also known as miR-595) in ESCC tissues and cells. **a** The circinteractome predicted a complementary site between the wild type of circ_NRIP1 (simplified as circ_NRIP1-wt) and miR-595. Circ_NRIP1-wt was mutated of this potential binding sequence, named as circ_NRIP1-mut. **b**, **c** Dual-luciferase reporter assay confirmed luciferase activity of KYSE30 and KYSE450 cells co-transfected with circ_NRIP1-wt/mut and mimics of miR-595 or negative control miRNA (simplified as miR-595 or miR-NC). **d** RT-qPCR detected miR-595 expression level in KYSE30 and KYSE450 cells introduced with si-circ_NRIP1 or si-NC. **e** RT-qPCR detected miR-595 expression level in HET-1A, KYSE30 and KYSE450 cells. **f** RT-qPCR detected miR-595 expression level in Tumor (n = 42) and Normal (n = 42) from ESCC patients. **g** Pearson’s correlation coefficient assay analyzed the correlation between circ_NRIP1 and miR-595 in ESCC tumor tissues (n = 42). *P < 0.05

The molecular regulatory mechanism of circ_NRIP1 was figured out, and target miRNAs were retrieved on bioinformatics tools. The circinteractome arithmetic (https://circinteractome.nia.nih.gov/api/v2/mirnasearch?circular_rna_query=hsa_circ_0004771&mirna_query=&submit=miRNA+Target+Search) provided a complementary site between circ_NRIP1 and miR-595; to further validate this hypothesis, circ_NRIP1- mut was also constructed according to these putative binding sites (Fig. 3a). Dual-luciferase reporter assay showed a significant decrease of luciferase activity of KYSE30 and KYSE450 cells co-transfected with circ_NRIP1-wt and miR-595 mimic (Fig. 3b, c); whereas, circ_NRIP1-mut failed to affect luciferase activity with miR-595 mimic introduction. In addition, si-circ_NRIP1 transfection led to endogenous miR-595 upregulation in KYSE30 and KYSE450 cells (Fig. 3d). These data prompted a target relationship between circ_NRIP1 and miR-595. Expression of miR-595 was lower in ESCC cells as well as tumor tissues (Fig. 3e, f). Moreover, Pearson’s correlation coefficient assay analyzed that miR-595 expression was corrected with circ_NRIP1 in ESCC patients (*r* = -0.7252, *P* < 0.0001; Fig. 3g).

### Blocking miR-595 abated the inhibitory effect of circ_NRIP1 knockdown on ESCC cell growth, migration and invasion in vitro

**Fig. 4:**
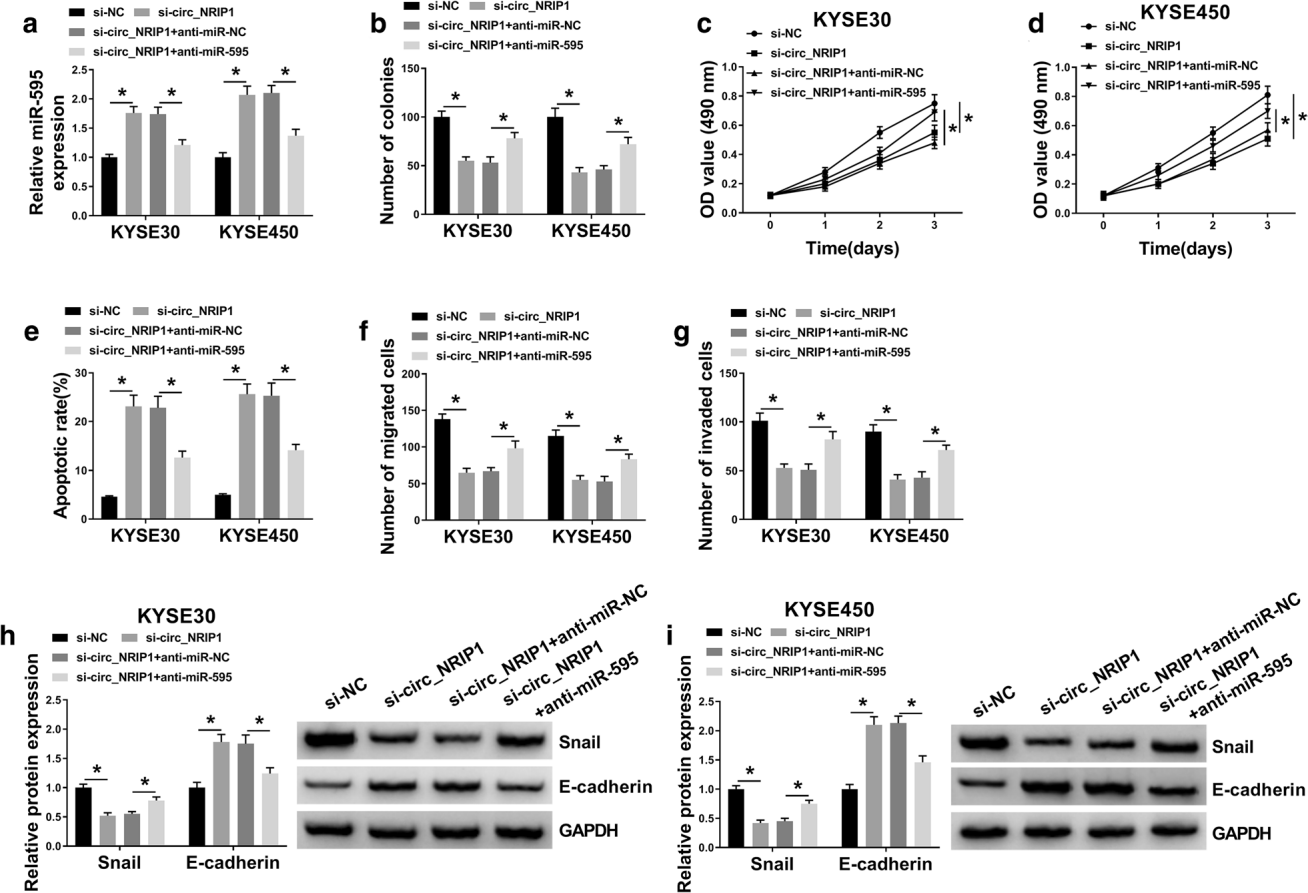
The reciprocal role of circ_NRIP1 and miR-595 in ESCC cells in vitro. **a** RT-qPCR detect miR-595 expression level in KYSE30 and KYSE450 cells administrated with si-NC alone, si-circ_NRIP1 alone or combined with anti-RNAs against miR-595 or negative control miRNA (named as anti-miR-595 or anti-miR-NC). **b** Number of colonies was measured by colony formation assay after transfection. **c**, **d** MTS assay determined OD value of transfected cells after transfection for 0–3 days. **e** Annexin V-FITC and PI double staining method and FCM analyzed apoptotic rate of transfected cells after transfection for 1 day. **f**, **g** Transwell assays evaluated numbers of migrated cells and invaded cells after transfection for 1 day. **h**, **i** Western blotting examined protein expression of snail and E-cadherin in transfected cells after transfection for 1 day. *P < 0.05

The reciprocal role of circ_NRIP1 and miR-595 in ESCC cells in vitro was following detected. KYSE30 and KYSE450 cells were alone administrated with si-NC or si-circ_NRIP1, or co-transfected with si-circ_NRIP1 and anti-miR-595 or anti-miR-NC. The upregulation of miR-595 in KYSE30 and KYSE450 cells was attenuated due to anti-miR-595 introduction (Fig. 4a). Moreover, the inhibition of circ_NRIP1 knockdown on colony formation and cell viability was diminished (Fig. 4b–d and Additional file 3: Figure S3a), whereas the increase of apoptotic rate was declined in the presence of anti-miR-595 (Fig. 4e and Additional file 3:: Figure S3d). Cell migration and invasion were inhibited by circ_NRIP1 silence, which was improved by miR-595 deficiency (Fig. 4f–g and Additional file 3: Figure S3b, c), accompanied with increased snail and decreased E-cadherin (Fig. 4h, i). These results demonstrated that blocking miR-595 could partially abated the suppressive role of circ_NRIP1 knockdown in ESCC cell growth, migration and invasion, suggesting a circ_NRIP1/miR-595 axis in ESCC cell malignant progression in vitro.

### SEMA4D was negatively regulated by miR-595 in ESCC via target binding

**Fig. 5:**
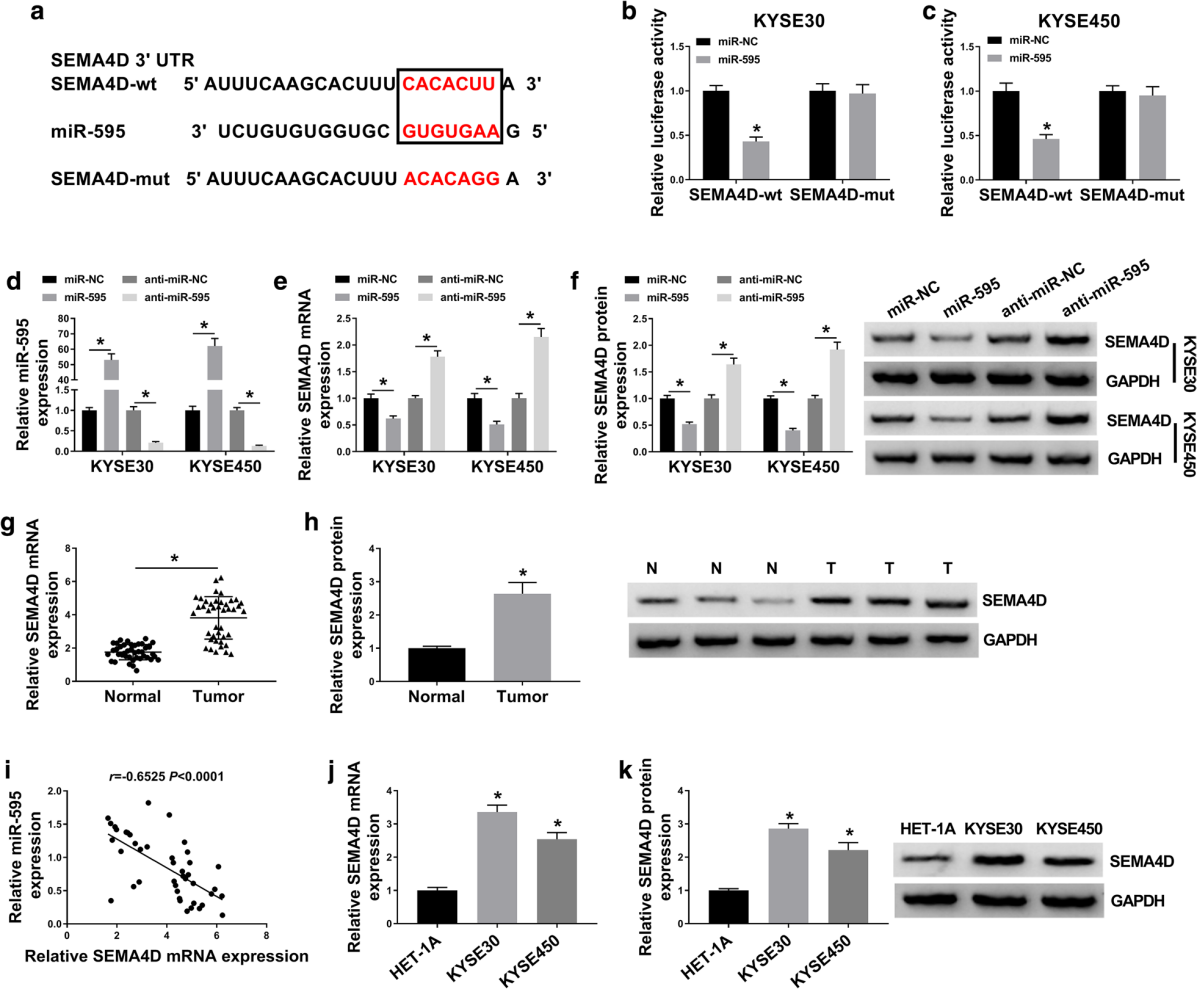
The expression of Semaphorin 4D (simplified as SEMA4D) in ESCC tissues and cells. **a** The miRDB predicted a complementary site between the wild type of SEMA4D 3′UTR (simplified as SEMA4D-wt) and miR-595. SEMA4D-wt was mutated of this potential binding sequence, named as SEMA4D-mut. **b**, **c** Dual-luciferase reporter assay confirmed luciferase activity of KYSE30 and KYSE450 cells co-transfected with SEMA4D-wt/mut and miR-595 or miR-NC. **d**–**f** RT-qPCR detected **d** miR-595 and **e** SEMA4D mRNA expression levels, and western blotting measured SEMA4D protein expression level in KYSE30 and KYSE450 cells introduced with miR-NC, miR-595, anti-miR-NC, or anti-miR-595. **g** RT-qPCR detected SEMA4D mRNA expression level in Tumor (n = 42) and Normal (n = 42) from ESCC patients. **h** Western blotting measured SEMA4D protein level in three paired tissues from ESCC patients. **i** Pearson’s correlation coefficient assay analyzed the correlation between miR-595 and SEMA4D mRNA expression in ESCC tumor tissues (n = 42). **j**, **k** RT-qPCR and western blotting detected SEMA4D expression levels in HET-1A, KYSE30 and KYSE450 cells. *P < 0.05

The miRDB arithmetic (http://mirdb.org/cgi-bin/target_detail.cgi?targetID=1297596) predicted a complementary site between miR-595 and SEMA4D 3′UTR (Fig. 5a); luciferase reporter assay further identified this potential binding in KYSE30 and KYSE450 cells, as declined luciferase activity of SEMA4D-wt when transfection with miR-595 mimic (Fig. 5b, c). Besides, miR-595 mimic transfection resulted in higher miR-595 and lower SEMA4D in KYSE30 and KYSE450 cells, and anti-miR-595 transfection caused lower miR-595 and higher SEMA4D (Fig. 5d–f). These data hinted a target relationship between miR-595 and SEMA4D. Expression of SEMA4D both on mRNA and protein levels was upregulated in ESCC tumor tissues (Fig. 5g, h). Moreover, there was a negative correlation between miR-595 and SEMA4D mRNA levels in ESCC patients according to Pearson’s correlation coefficient assay (*r* = -0.6525, *P* < 0.0001; Fig. 5i). SEMA4D expression levels were increased in ESCC cells as well (Fig. 5j, k). These data indicated a direct relationship of miR-595 and SEMA4D in ESCC, suggesting a possible reciprocal role of both in ESCC cells.

### Restoring SEMA4D abrogated the suppressive role of miR-595 in ESCC cell growth, migration and invasion in vitro

**Fig. 6:**
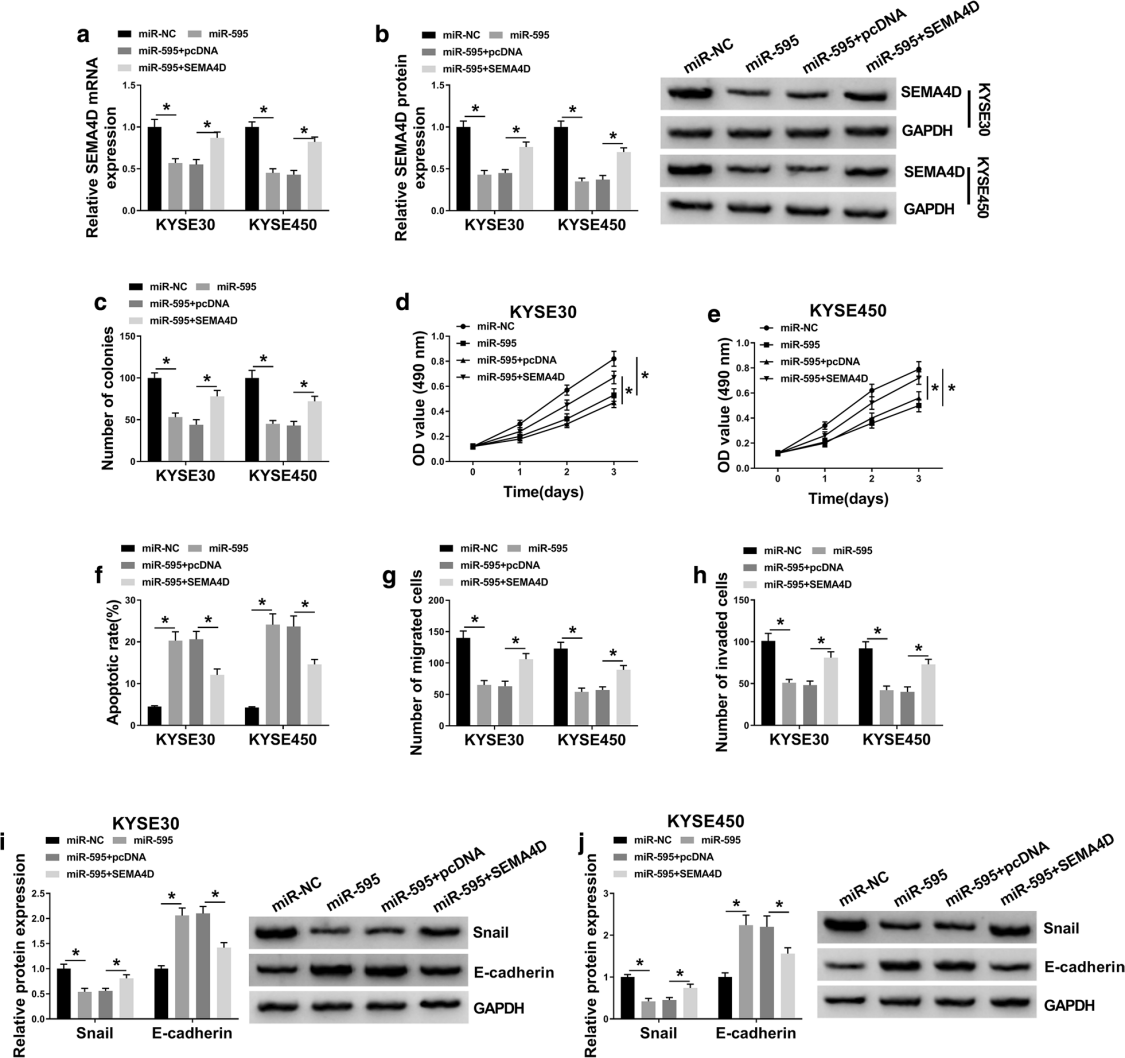
The reciprocal role of miR-595 and SEMA4D in ESCC cells in vitro. **a**, **b** RT-qPCR and western blotting detect SEMA4D expression levels in KYSE30 and KYSE450 cells administrated with miR-NC alone, miR-595 alone or combined with pcDNA3.1(-) vectors carrying SEMA4D or not (named as SEMA4D or pcDNA) **c** Number of colonies was measured by colony formation assay after transfection. **d**, **e** MTS assay determined OD value of transfected cells after transfection for 0–3 days. **f** Annexin V-FITC and PI double staining method and FCM analyzed apoptotic rate of transfected cells after transfection for 1 day. **g**, **h** Transwell assays evaluated numbers of migrated cells and invaded cells after transfection for 1 day. **i**, **j** Western blotting examined protein expression levels of snail and E-cadherin in transfected cells after transfection for 1 day. *P < 0.05

KYSE30 and KYSE450 cells were administrated with miR-NC mimic alone or miR-595 mimic alone, or together with SEMA4D-overexpression vector and pcDNA vector. The miR-595 mimic transfection decreased endogenous SEMA4D expression level in KYSE30 and KYSE450 cells, and this downregulation was diminished due to SEMA4D-overexpression vector introduction (Fig. 6a, b). The miR-595 mimic suppressed cell growth of KYSE30 and KYSE450 cells, which was abolished by SEMA4D restoration, as evidenced by elevation of colony number and OD values (Fig. 6c–e and Additional file 4: Figure S4a), as well as reduction of apoptotic rate (Fig. 6f and Additional file 4: Figure S4d). Cell migration and invasion were inhibited by miR-595 overexpression, which was counteracted by SEMA4D upregulation, as depicted by promoted numbers of migrated cells and invaded cells (Fig. 6g–h and Additional file 4: Figure S4b, c), as well as increased snail expression and decreased E-cadherin expression (Fig. 6i, j). These results demonstrated that miR-595 overexpression could reverse ESCC cell growth, migration and invasion in vitro by upregulating SEMA4D, suggesting a miR-595/SEMA4D axis in ESCC cell malignant progression.

### Knockdown of circ_NRIP1 inhibited PI3K/AKT signaling pathway in ESCC cells by circ_NRIP1/miR-595/SEMA4D axis

**Fig. 7:**
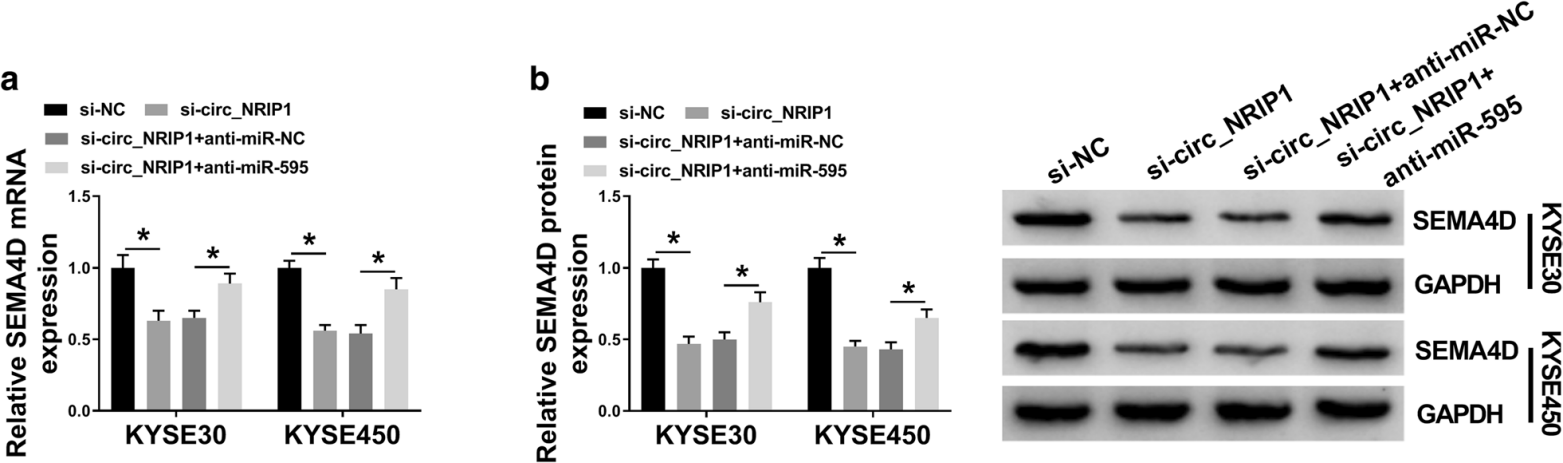
The regulatory effect of circ_NRIP1 on SEMA4D in ESCC cells. **a**, **b** RT-qPCR and western blotting detected SEMA4D expression levels in KYSE30 and KYSE450 cells administrated with si-NC alone, si-circ_NRIP1 alone or combined with anti-miR-595 or anti-miR-NC. *P < 0.05

**Fig. 8:**
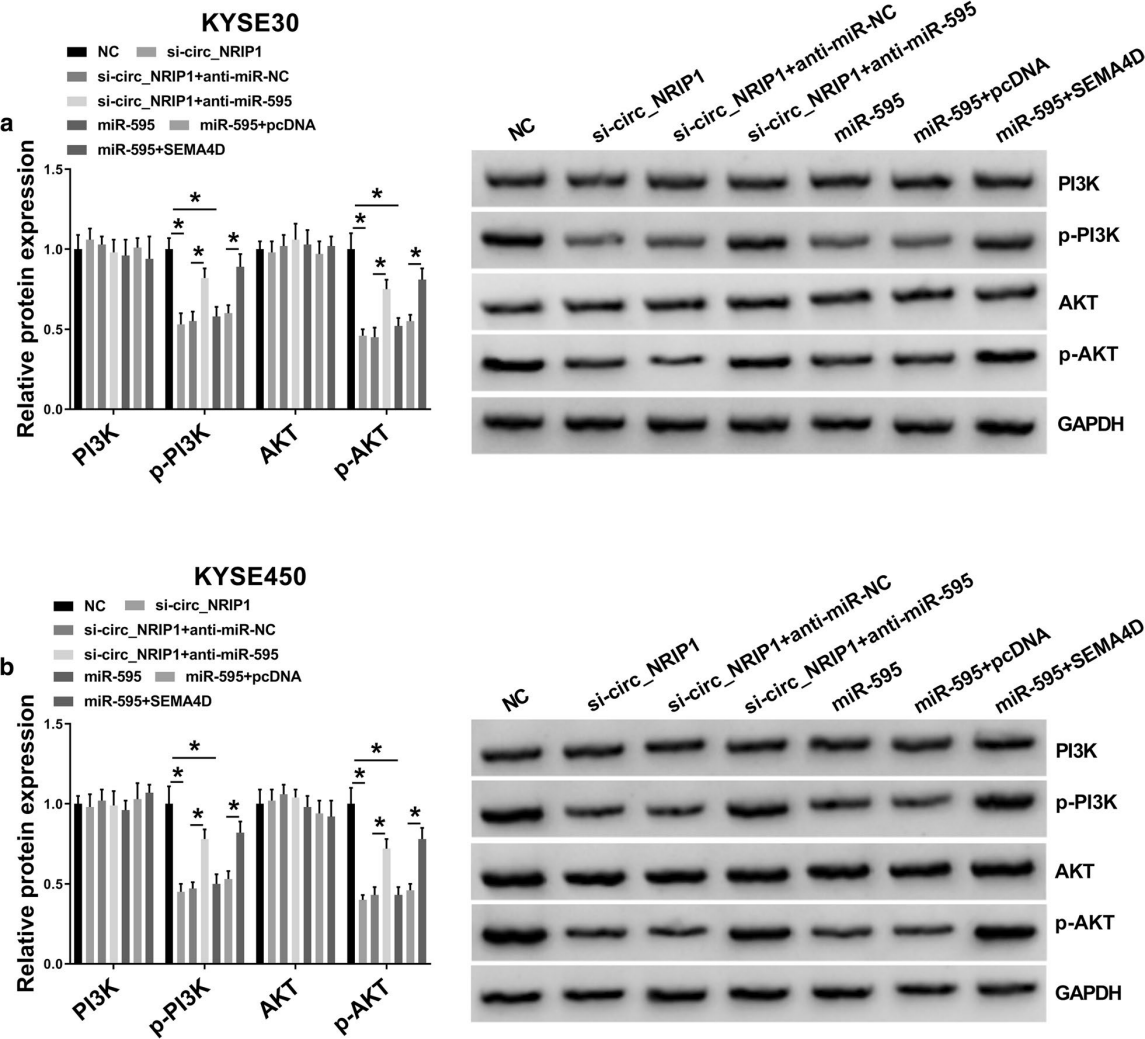
The role of circ_NRIP1, miR-595 and SEMA4D in phosphatidylinositol-3-hydroxykinase (PI3K)/AKT pathway in ESCC cells. **a**, **b** Western blotting examined protein expression levels of PI3K, AKT and phosphorylated PI3K and AKT (called as p-PI3K and p-AKT) in KYSE30 and KYSE450 cells administrated with si-NC combined with miR-NC (simplified as NC), si-circ_NRIP1 alone or together with anti-miR-NC or anti-miR-595, miR-595 alone or together with pcDNA or SEMA4D. *P < 0.05

By monitoring SEMA4D expression in KYSE30 and KYSE450 cells administrated with si-circ_NRIP1 alone or combined with anti-miR-595, we observed that circ_NRIP1 knockdown could indirectly downregulate SEMA4D expression levels via miR-595 (Fig. 7a, b). Thus, abovementioned findings showed a circ_NRIP1/miR-595/SEMA4D axis in ESCC cell malignant progression. Furthermore, the activities of PI3K and AKT were both inhibited by circ_NRIP1 knockdown and/or miR-595 overexpression compared with negative control cells (co-transfected with si-NC and miR-NC mimic, as reflected by the decrease of p-PI3K and p-AKT (Fig. 8a, b). By the way, there was no significant difference of circ_NRIP1 and miR-595 expression levels among groups with si-NC alone transfection, miR-NC mimic alone transfection and co-transfection of si-NC and miR-NC mimic (Figure S2). The inhibition of circ_NRIP1 silence and miR-595 upregulation was separately reversed by silencing miR-595 and facilitating SEMA4D (Fig. 8a, b). Therefore, PI3K/AKT signaling pathway was inactivated by circ_NRIP1 knockdown via regulating circ_NRIP1/miR-595/SEMA4D axis.

### Silencing circ_NRIP1 retarded tumor growth of ESCC cells in vivo, accompanied with miR-595 upregulation and SEMA4D downregulation

**Fig. 9:**
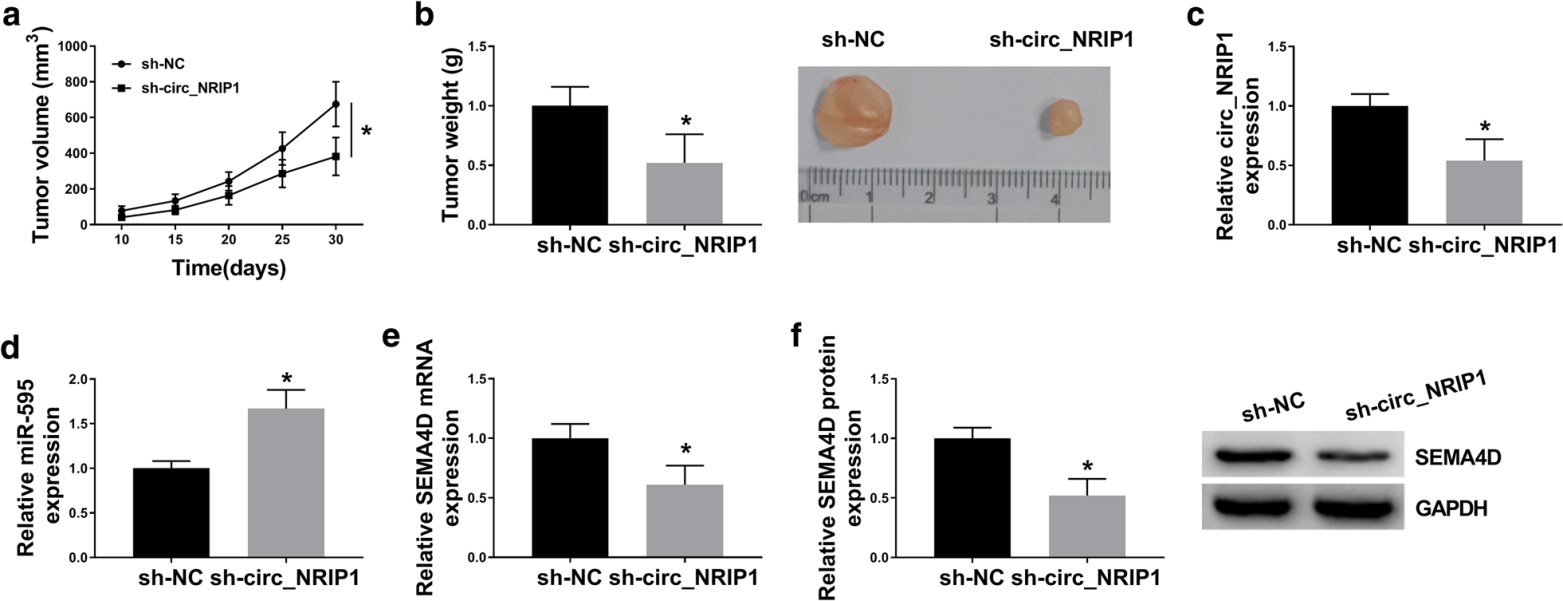
The role of circ_NRIP1 in tumor growth of ESCC cells in vivo. **a** Tumor volume was measured every five days after implantation of KYSE450 cells stably expressed shRNA against circ_NRIP1 or negative control sequences (simplified as sh-circ_NRIP1 or sh-NC) for 10 days. **b** Tumor weight was recorded on the last day. **c**–**f** RT-qPCR detected **c** circ_NRIP1, **d** miR-595, **e** SEMA4D mRNA levels, and western blotting measured **f** SEMA4D protein level in tumor tissues from xenograft mice. *P < 0.05

The in vivo role of circ_NRIP1 in cell growth was further surveyed. With sh-circ_NRIP1 transfection, cell growth of KYSE450 cells in nude mice was restrained comparing to sh-NC transfection, as described by lowered tumor volume and weight (Fig. 9a, b). Furthermore, the expression level of circ_NRIP1 was knocked down in xenograft tumor tissues (Fig. 9C), as accompanied with higher miR-595 and lower SEMA4D (Fig. 9d–f). These data indicated a similar suppressive role of circ_NRIP1 silence in tumor growth in vivo probably via miR-595/SEMA4D axis.

## Discussion

To date, circRNA microarray and RNA sequencing were popular high-throughput screening techniques to survey the differently expressed circRNAs, and a dozen of circRNAs had been reported to be upregulated and thus promoted ESCC progression. For example, Pan et al. [22] found that a novel circRNA hsa_circ_0006948 was increased in tissues of human ESCC tumors, and its upregulation promoted cell proliferation, migration and invasion of ESCC cells both in vitro and in vivo. Moreover, miR-490-3p/HMGA2 axis was validated to be underlying the tumor-suppressive role of hsa_circ_0006948 in ESCC [22]. Here, we noticed circ_NRIP1 was a recently identified circRNA and acted as a natural ceRNA for miRNAs in cancers [12, 13]. Therefore, we explored the role of circ_NRIP1 in colony formation, cell viability, apoptosis, migration, and invasion in ESCC cells in vitro, and tumor growth in vivo through serving as a miRNA sponge.

In this study, we discovered an upregulation of circ_NRIP1 in tumor tissues of ESCC patients and cell lines, which was similar to the finding of Huang et al*.* [13]. In that research, they validated higher expression of circ_NRIP1 (namely hsa_circ_0004771) in both tissues and plasma of ESCC patients. Functionally, we observed that silencing of circ_NRIP1 could suppress colony formation, cell viability, migration, and invasion of KYSE30 and KYSE450 cells, accompanied with apoptosis rate promotion. Furthermore, tumor growth of KYSE450 cells in xenograft mice was retarded by circ_NRIP1 silence, as well. Among these effects, cell proliferation inhibition of circ_NRIP1 in ESCC cells both in vitro and in vivo was also illuminated before [13]. Similarly, we and Huang et al. [13] confirmed circ_NRIP1 as a sponge for miRNAs including miR-595 and miR-339-5p. Moreover, we also noticed an inhibition of circ_NRIP1 knockdown on PI3K and AKT activities, which was previously disclosed in renal carcinoma cells [23]. However, this study was probably the first evidence that circ_NRIP1 inactivated PI3K/AKT signaling pathway in ESCC. By the way, the association between circ_NRIP1 and clinicopathological characteristics such as prognosis [13] was not further monitored. Even though circ_NRIP1 was clarified as one of top upregulated circRNA in ESCC, colorectal cancer and breast cancer, Li et al. [24] observed top ten reduced circRNAs including circ_NRIP1 in gastric cancer patients’ plasma, and this probably was the rare research announcing upregulation of circ_NRIP1 in cancer-related patients. These outcomes might support a tissue-specificity of circ_NRIP1 expression, suggesting that the emerging research of circ_NRIP1 in human cancers was still nascent.

MiR-595 was also declared to play a dual-role in human cancers via functioning as an oncogene or tumor suppressor [19]. Functionally, miR-595 was a phenotypic regulator of cell proliferation [19, 20], metastasis [20, 25], autophagy [26], and chemosensitivity [27, 28]. Besides, it was indicated as a significant and independent indicator of poor prognosis in epithelial ovarian cancer [29], and one independent factor of diagnosis in hepatocellular carcinoma [30]. Nevertheless, it was still vague about how miR-595 itself was regulated. Here, we identified that miR-595 could be modulated by circ_NRIP1 in ESCC via competing binding. Moreover, the expression and role of miR-595 in ESCC cells were also clarified. Expression of miR-595 was downregulated in tumor tissues of ESCC patients and cell lines, prompting a potential anti-tumor role in ESCC. Gain-of-function experiments demonstrated an inhibitory effect of miR-595 overexpression on colony formation, cell viability, migration, and invasion in KYSE30 and KYSE450 cells. Notably, multiple miRNAs were declared to be vital regulators of PI3K/AKT signaling pathway in ESCC, such as miR-203a [31], miR-30d [32], and miR-214 [33]. However, it was unclear about the role of miR-595 in PI3K/AKT signal in ESCC. Thus, we uncovered an inhibition of miR-595 on PI3K and AKT activities.

SEMA4D was an oncogene and contributed to tumor progression in human patients or experimental models [21]. Targeting SEMA4D had been ongoing at early-stage clinical trials, such as in neuroendocrine pancreatic cancer [34, 35]. In ESCC, Wang et al. [36] very recently unveiled that SEMA4D was upregulated in ESCC, and its knockdown could decrease cell viability, migration and invasion of ESCC KYSE-150 and TE-10 cells, accompanied with apoptosis promotion. Luckily, our data supported the upregulation of SEMA4D in ESCC tumor tissues and cells. We further defined a tumor-promoting effect of SEMA4D recovery on cell viability, colony formation, migration and invasion of KYSE30 and KYSE450 cells. Interestingly, there were only several miRNAs had been announced to affect SEMA4D expression at post transcription level, including miR-4319 and miR-214 [36, 37]. Here, we provided miR-595 as a new epigenetic regulator of SEMA4D; In addition, SEMA4D downregulation mediated by circ_NRIP1 knockdown and/or miR-595 overexpression was hidden in the inactivation of PI3K/AKT signaling pathway; restoring SEMA4D could attenuate the suppression on PI3K/AKT signal, which was analogous to previous finding in bladder cancer cells [38].

## Conclusion

**Fig. 10:**
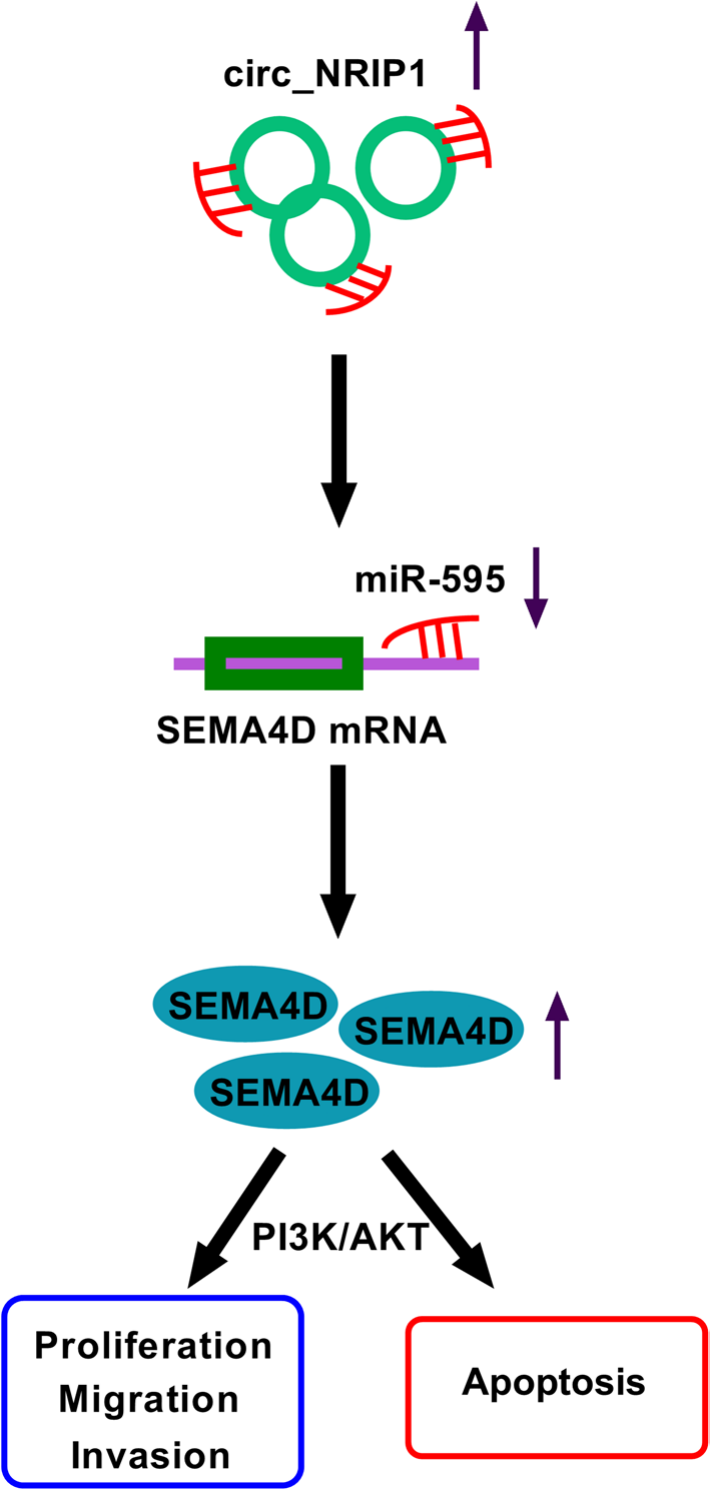
The schematic diagram of circ_NRIP1/miR-595/SEMA4D axis in ESCC cell progression. Circ_NRIP1 sponged miR-595 to guide SMEA4D expression in regulation of ESCC cell proliferation, migration, invasion, apoptosis, and PI3K/AKT signal

In conclusion, this study demonstrated an oncogenic role of circ_NRIP1 in ESCC cells by suppressing cell growth, migration and invasion both in vitro and in vivo via circ_NRIP1/miR-595/SEMA4D axis and PI3K/AKT signaling pathway (Fig. 10). Our data might contribute to circ_NRIP1 being a potential but vital target in the diagnosis and treatment of ESCC.

## Supplementary Information

**Additional file 1: Figure S1.** Expression of circ_NRIP1 in human ESCC cells. RT-qPCR detected relative circ_NRIP1 expression in human ESCC cell lines (KYSE30 and KYSE450) and normal HET-1A cell line. *P < 0.05.

**Additional file 2: Figure S2.** Expression of circ_NRIP1 and miR-595 in ESCC cells transfected with negative control oligonucleotides. **a**, **b** RT-qPCR measured levels of circ_NRIP1 and miR-595 in KYSE30 and KYSE450 cells transfected with si-NC alone, miR-NC mimic (miR-NC) alone, or si-NC together with miR-NC (simplified as NC). n.s. presented no significant difference among these groups.

**Additional file 3: Figure S3.** The reciprocal role of circ_NRIP1 and miR-595 in ESCC cells in vitro. **a** Number of colonies was measured by colony formation assay, **b**, **c** transwell assays evaluated numbers of migrated cells and invaded cells, and **d** FCM analyzed apoptotic rate after transfection.

**Additional file 4: Figure S4.** The reciprocal role of miR-595 and SEMA4D in ESCC cells in vitro. **a** Number of colonies was measured by colony formation assay after transfection, **b**, **c** transwell assays evaluated numbers of migrated cells and invaded cells, and (D) FCM analyzed apoptotic rate after transfection.

## Data Availability

The datasets used and/or analyzed during the current study are available from the corresponding author on reasonable request.

## Abbreviations

ESCC: Esophageal squamous cell carcinoma
NRIP1: Nuclear receptor interacting protein 1
circRNAs: Circular RNAs
circ_NRIP1: CircRNA NRIP1
miR-595: MicroRNA-595-5p
SEMA4D: Semaphorin 4D
PI3K: Phosphatidylinositol-3-hydroxykinase
ncRNAs: Noncoding RNAs
ceRNAs: Competitive endogenous RNAs
FBS: Fetal bovine serum
JCRB: Japanese Collection of Research Bioresources
RT-qPCR: Reverse transcription-quantitative polymerase chain reaction
GAPDH: Glyceraldehyde-phosphate dehydrogenase
U6: U6 snoRNA
si-circ_RIP1: SiRNA against circ_NRIP1
sh-circ_NRIP1: ShRNA against circ_NRIP1
anti-miR-595: Anti-RNAs against miR-595
OD: Optical density
FITC: Fluorescein isothiocyanate
PI: Propidium iodide
3′UTR: 3′ Untranslated region
wt: Wild type
mut: Mutant type
