# Supplementary figures and images for: circ_NRIP1 is oncogenic in malignant development of esophageal squamous cell carcinoma (ESCC) via miR-595/SEMA4D axis and PI3K/AKT pathway
Source: Cancer Cell Int. 2021 May 6;21:250. doi: 10.1186/s12935-021-01907-x (PMC8101145; doi:10.1186/s12935-021-01907-x)

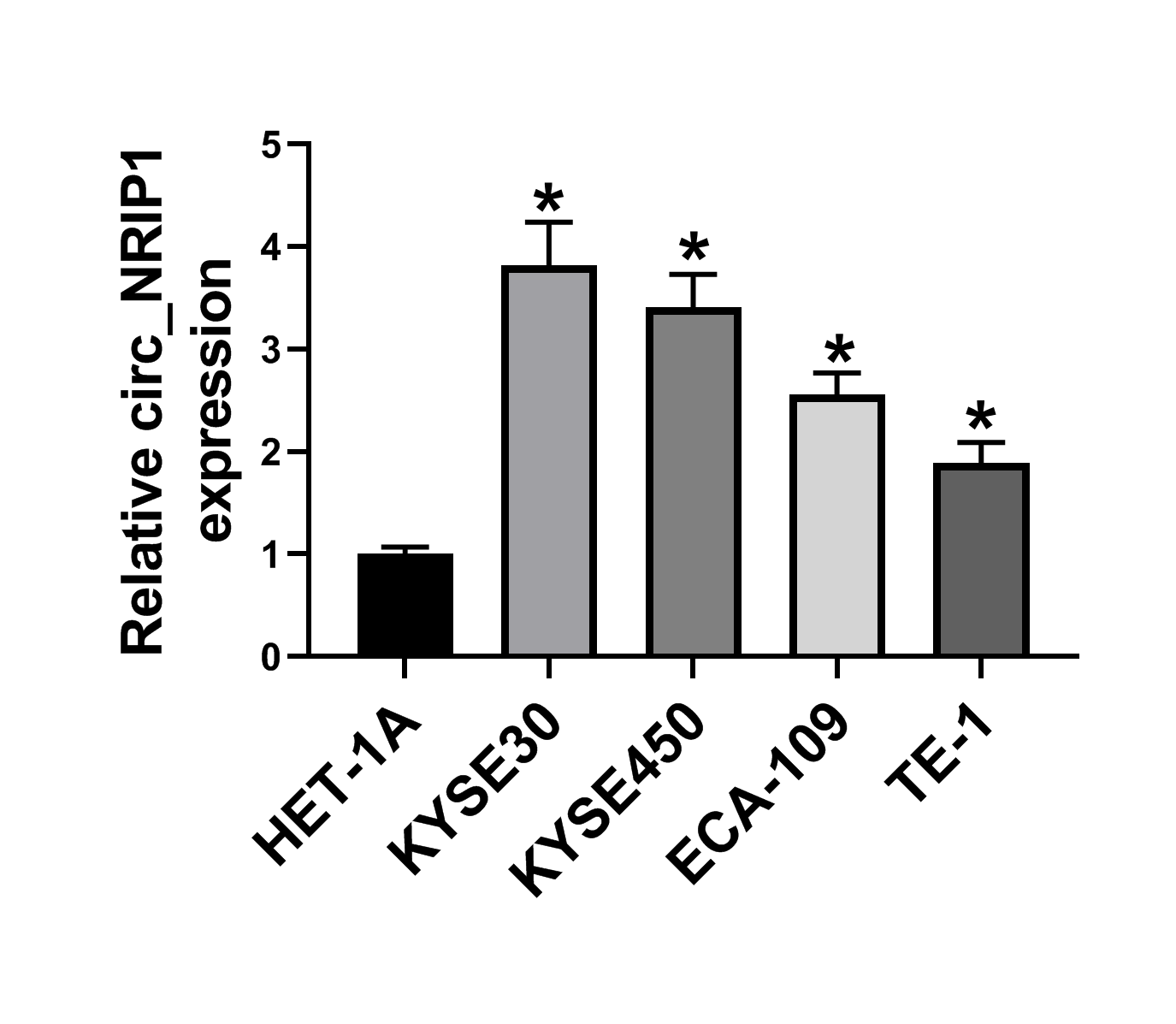

Supplement: Supplementary file 1 — Additional file 1: Figure S1. Expression of circ_NRIP1 in human ESCC cells. RT-qPCR detected relative circ_NRIP1 expression in human ESCC cell lines (KYSE30 and KYSE450) and normal HET-1A cell line. *P < 0.05. [file 12935_2021_1907_MOESM1_ESM.tif]

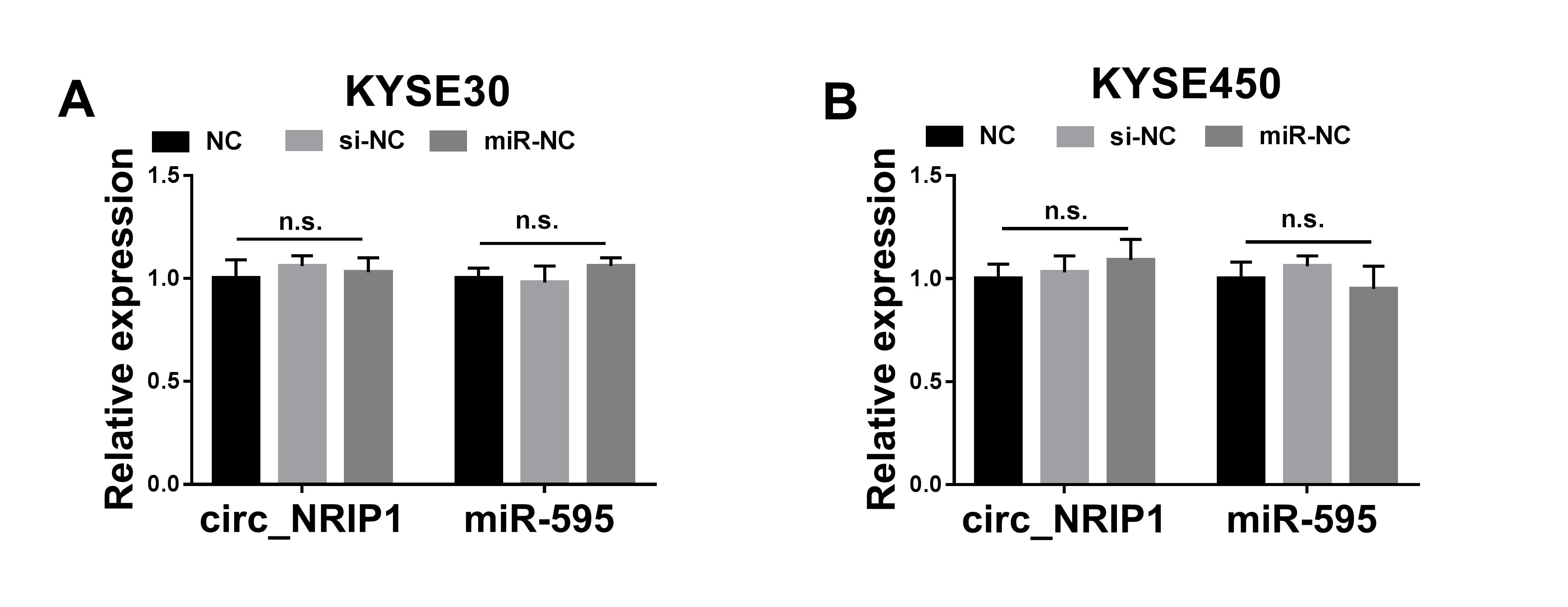

Supplement: Supplementary file 2 — Additional file 2: Figure S2. Expression of circ_NRIP1 and miR-595 in ESCC cells transfected with negative control oligonucleotides. a, b RT-qPCR measured levels of circ_NRIP1 and miR-595 in KYSE30 and KYSE450 cells transfected with si-NC alone, miR-NC mimic (miR-NC) alone, or si-NC together with miR-NC (simplified as NC). n.s. presented no significant difference among these groups. [file 12935_2021_1907_MOESM2_ESM.tif]

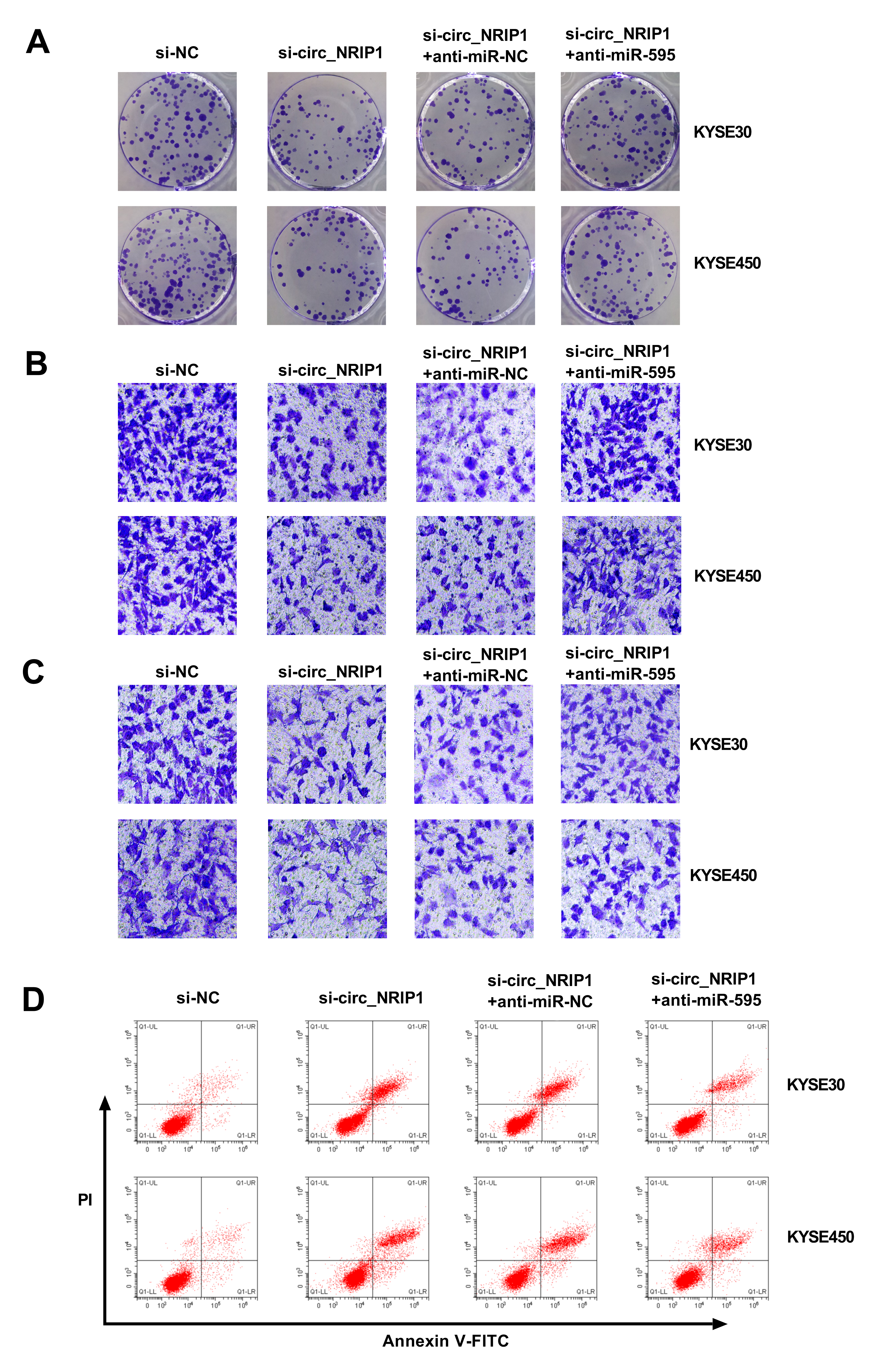

Supplement: Supplementary file 3 — Additional file 3: Figure S3. The reciprocal role of circ_NRIP1 and miR-595 in ESCC cells in vitro. a Number of colonies was measured by colony formation assay, b, c transwell assays evaluated numbers of migrated cells and invaded cells, and d FCM analyzed apoptotic rate after transfection. [file 12935_2021_1907_MOESM3_ESM.tif]

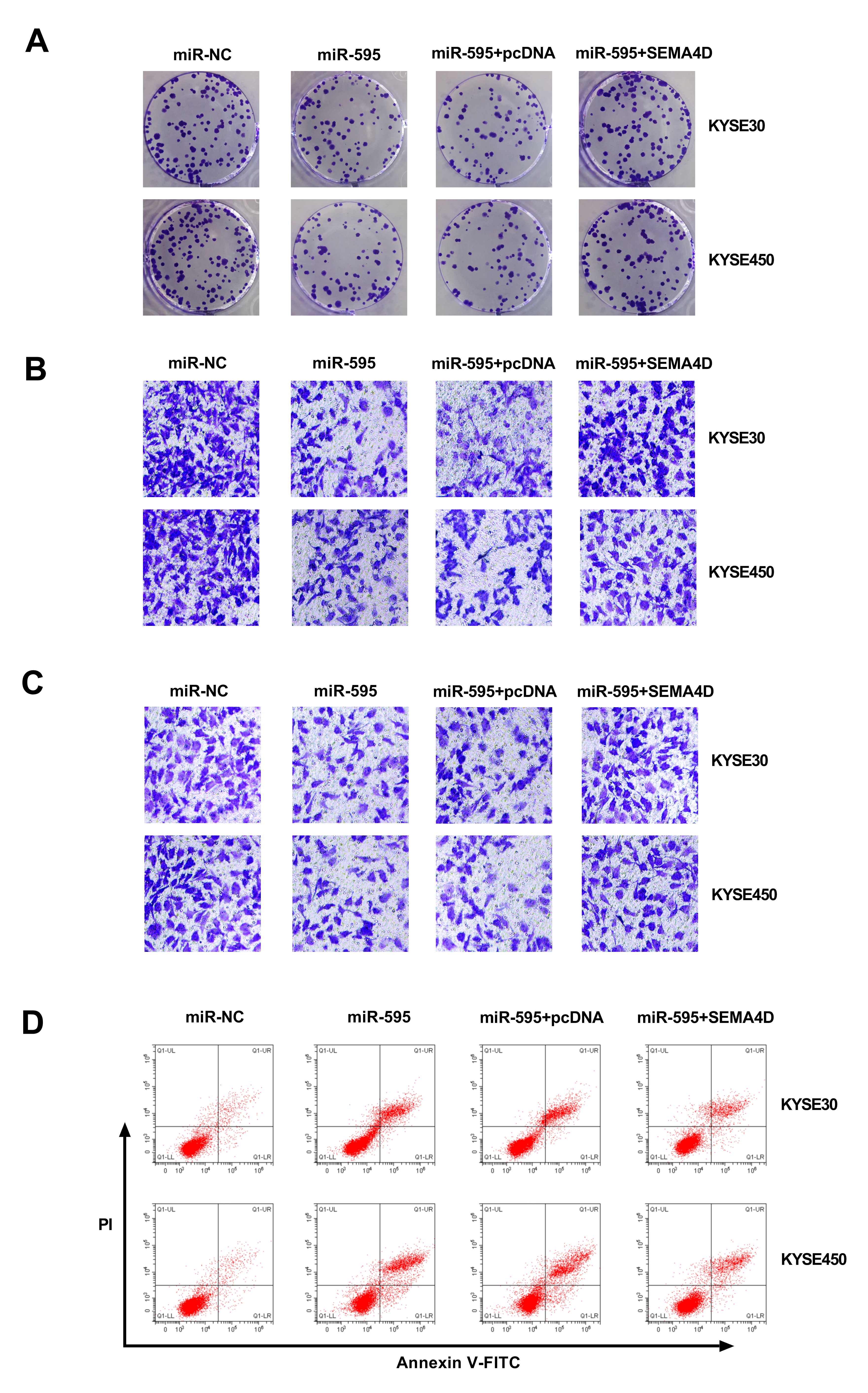

Supplement: Supplementary file 4 — Additional file 4: Figure S4. The reciprocal role of miR-595 and SEMA4D in ESCC cells in vitro. a Number of colonies was measured by colony formation assay after transfection, b, c transwell assays evaluated numbers of migrated cells and invaded cells, and (D) FCM analyzed apoptotic rate after transfection. [file 12935_2021_1907_MOESM4_ESM.tif]
